# Parasite Load and STRs Genotyping of *Toxoplasma gondii* Isolates From Mediterranean Mussels (*Mytilus galloprovincialis*) in Southern Italy

**DOI:** 10.3389/fmicb.2020.00355

**Published:** 2020-03-05

**Authors:** Mario Santoro, Maurizio Viscardi, Federica Boccia, Giorgia Borriello, Maria Gabriella Lucibelli, Clementina Auriemma, Aniello Anastasio, Vincenzo Veneziano, Giorgio Galiero, Loredana Baldi, Giovanna Fusco

**Affiliations:** ^1^Department of Integrative Marine Ecology, Stazione Zoologica Anton Dohrn, Naples, Italy; ^2^Istituto Zooprofilattico Sperimentale del Mezzogiorno, Portici, Italy; ^3^Department of Veterinary Medicine and Animal Productions, Università degli Studi di Napoli Federico II, Naples, Italy

**Keywords:** *Toxoplasma gondii*, toxoplasmosis, food borne zoonoses, Mediterranean mussel, *Mytilus galloprovincialis*

## Abstract

Toxoplasmosis is a zoonotic food-borne disease caused by *Toxoplasma gondii*, a land-derived protozoan parasite that infects a broad range of terrestrial and aquatic hosts. *T. gondii* may reach coastal waters via contaminated freshwater runoff and its oocysts may enter into the marine food web. Marine invertebrates as mussels being filter feeders are exposed and may concentrate *T. gondii* oocysts representing a potential source of infection for animals and humans. The present works investigated the prevalence, parasite burden and genotypes of *T. gondii* in the Mediterranean mussels (*Mytilus galloprovincialis*) from southern Italy. We sampled a total of 382 individual Mediterranean mussels from May to August 2018 from seven production sites in the Gulf of Naples (Campania region). An additional sample including 27 farmed Mediterranean mussels was obtained in February 2018 from a mollusk depuration plant in Corigliano Calabro (Calabria region). *T. gondii* DNA was detected in 43 out of 409 (10.5%) Mediterranean mussels from seven out of eight sampling sites. The number of *T. gondii* copies/g in the digestive gland ranged from 0.14 to 1.18. Fragment analysis of Short Tandem Repeats (STRs) at 5 microsatellite loci was performed from 10 *T. gondii* PCR positive samples revealing the presence of five distinct genotypes including one corresponding to type I and four atypical genotypes. These findings suggest potential implications of epidemiological importance for human and animal health because both type I and atypical genotypes could be highly pathogenic.

## Introduction

Toxoplasmosis is a food-borne zoonotic disease caused by *Toxoplasma gondii*, an obligate intracellular protozoan parasite with a global distribution and great ability to infect a broad range of hosts. Its life cycle includes three infectious stages: tachyzoites, free parasites in host fluids and infecting host cells in the acute stage; bradyzoites, occurring in tissue cysts, and sporozoites contained inside the oocysts shed with the feces to the environment by felids (definitive hosts). The major routes of *T. gondii* infection to humans include the accidental ingestion of sporulated oocysts in water, fruits or vegetables, and consumption of raw and undercooked meat containing tissue cysts of the parasite (Mead et al., 1999; Pereira et al., 2010; Robert-Gangneux and Dardè, 2012).

Three predominant archetypal lineages of *T. gondii* were originally described (named types I, II, and III). More recently, six clades have been characterized, and diverse atypical genotypes have been also described by sequence-based analyses (Ajzenberg et al., 2004; Lehmann et al., 2006; Sibley et al., 2009; Robert-Gangneux and Dardè, 2012). Several studies support the hypothesis that *T. gondii* genotypes may be related to different disease severities in both animals and humans (see Robert-Gangneux and Dardè, 2012).

Special interest has recently been focused on the spillover of pathogens from regional terrestrial animals into the aquatic ecosystem since many typical terrestrial pathogens of zoonotic interest have been found in different classes of marine organisms increasing the reports of lethal cases of infection in marine mammals (Miller et al., 2004; VanWormer et al., 2014; Grattarola et al., 2016; Aguirre et al., 2019; Santoro et al., 2019a).

Aquatic invertebrates may significantly influence waterborne transport of *T. gondii*, by enhanced settling and subsequent benthos concentration, and by facilitating ingestion by invertebrate vectors (Mazzillo et al., 2013; Aguirre et al., 2019). Shellfish being filter feeders are exposed and may concentrate a variety of pathogens that via freshwater runoff from regional terrestrial environment flow into the marine ecosystem (Arkush et al., 2003; VanWormer et al., 2014). The consumption of shellfish has been shown to be a risk factor for acquiring *Toxoplasma* infection in animals and humans (Conrad et al., 2005; Jones et al., 2009). *T. gondii* oocysts can remain viable for at least 2 years in seawater, and at least 21 days after internalization by Mediterranean mussels (*Mytilus galloprovincialis*) (Arkush et al., 2003; Lindsay and Dubey, 2009).

The Mediterranean mussel is the most economically important shellfish produced and consumed in the Western Mediterranean. The Gulf of Naples is among the most important production sites of the Mediterranean mussel in southern Italy with about 4170 tons per year (Prioli, 2008). In the Mediterranean basin, the occurrence and quantification of *T. gondii* DNA in Mediterranean mussels has been demonstrated from Italy and Turkey (Aksoy et al., 2014; Marangi et al., 2015; Tedde et al., 2019), however, data on genotyping exists only from the western coast of the Antolian Peninsula (Turkey) where *T. gondii* type I was the only genotype detected (Aksoy et al., 2014). The present study aimed to report the prevalence, parasite burden and genotypes of *T. gondii* detected in samples of Mediterranean mussel from southern Italy.

## Materials and Methods

### Study Area and Sampling Sites

The Gulf of Naples (40° 44′ 4.19″ N - 14° 16′ 18.60″ E) is a natural semi-enclosed basin located over the continental shelf in the south-eastern Tyrrhenian Sea (Western Mediterranean Sea) of the Campania region. The shoreline region of the Gulf of Naples is a densely populated area characterized by the presence of industrial and harbor activities and commercial discharges. Its water quality is also strongly affected by artificial and natural river runoff as well as urban and agricultural activities.

**FIGURE 1:**
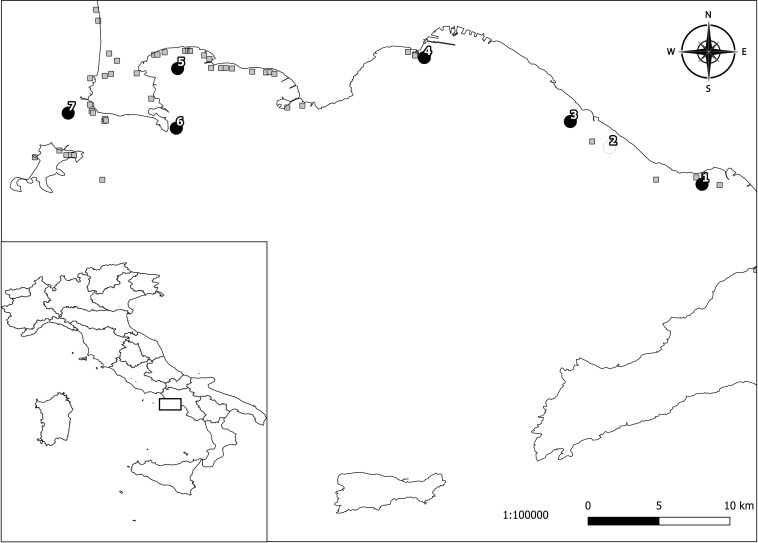
Sampling locations of Mediterranean mussels tested for *Toxoplasma gondii* in the Gulf of Naples (southern Italy): 1, Torre Annunziata; 2, Torre del Greco; 3, Ercolano; 4, Mergellina; 5, Lucrino; 6, Capo Miseno; 7, Monte di Procida. Black circles represent the localities where *T. gondii* positive samples were obtained. The white circle represents the locality where negative samples were obtained. The gray squares represent the known freshwater runoffs.

From May to August 2018, a total of 382 individual Mediterranean mussels (>5 cm in length) was collected from seven production sites in the Gulf of Naples (Figure 1). This sample size was calculated using the formula proposed by Thrusfield (2007) for a theoretically infinite population inserting the following values: expected prevalence (50%), confidence interval (95%) and desired absolute precision (5%). The Mediterranean mussels sampled were collected from 10% of the rows ready for harvest. The sampling sites and the known freshwater runoffs from the Gulf of Naples are presented in Figure 1. An additional sample including 27 farmed Mediterranean mussels (>5 cm in length) was obtained in February 2018 from a mollusk depuration plant in Corigliano Calabro (Cosenza province, Calabria region, southern Italy). This latter batch came from Porto Tolle (Rovigo province, Veneto region, northern Italy) and it had arrived at the mollusk depuration plant in Corigliano Calabro the month before.

### DNA Extraction

Immediately after sampling, the mussels were stored at +4°C, and within 12 h the digestive gland of each mussel was dissected and analyzed individually for *T. gondii* detection. For each mussel, 25 mg of the digestive gland was homogenized by tissue lyser (Qiagen) in sterile PBS buffer with two glass beads. Using 200 μl of the homogenate, automated extraction of nucleic acid was performed using the QIAsymphony DSP Virus/Pathogen mini kit (Qiagen, Hilden, Germany) according to the manufacturer’s protocol. As reference material, genomic DNA from *T. gondii* was obtained from the American Type Culture Collection (ATCC 50174D LGC Standards Italy).

### Standard Curve Generation

A 193 bp fragment of *T. gondii* B1 gene generated by PCR with the primers TOXO1 and TOXO2 was cloned into pGEM-T plasmid (pGEM-T Vector System I kit, Promega Corporation Madison United States) and propagated in a JM109 *Escherichia coli* strain of high efficiency chemical competent cells, according to manufacturer’s instructions (Lin et al., 2000). Plasmid DNA was purified using a Qiaprep Spin Miniprep Kit (Qiagen, Hilden, Germany); DNA integrity of the target was verified by capillary electrophoresis D5000 screen tape and reagent (Agilent Technologies, Santa Clara, CA, United States) on 2200 Tapestation stations (Agilent Technologies, Santa Clara, CA, United States), and confirmed by sequencing. The concentration of extract was measured by Qubit Fluorometer by Qubit dsDNA HS Assay kits (Life Technologies, Eugene, Oregon). The plasmid copy number was calculated considering that the plasmid size (including the insert) was 3179 bps using the DNA/RNA Copy Number Calculator^1^. The appropriate dilutions were performed in order to produce aliquots of 10^10^ copies of target DNA/10 μl of templates and frozen at −80°C for one use only. To generate the real-time PCR standard curves, 10-fold serial dilution of the *T. gondii* standard plasmid DNA, ranging from 10^1^ to 10^9^ copies of DNA/10 μl, was prepared for molecular quantifications. A standard curve was obtained by linear regression analysis of the threshold cycle (Ct) value (*y* axis) versus the log of the initial copy number present in each sample dilution (*x* axis). PCR efficiency (E) was calculated as E = 10 (1/slope)-1 (Lin et al., 2000; Peirson et al., 2003; Rutledge and Còté, 2003; Santoro et al., 2019b). The number of oocysts of *T. gondii* for each sample was estimated as suggested previously (Lass et al., 2012).

### Real-Time PCR

The real-time PCR (qPCR) for detection and quantification of *T. gondii* B1 gene was performed following a previous study (Lin et al., 2000), with modification of the final volume to 25 μl (Santoro et al., 2019b). In brief, 5 μl of template DNA was added to a reaction mixture containing 12.5 μl of 2X PCR universal master mix (Thermo Fischer Scientific, Waltham, MA, United States), 2.5 μl of the forward primer TOXO-F (5 μM, 5′-TCCCCTCTGCTGGCGAAAAGT-3′) (IDT, Coralville, IA, United States), 2.5 μl of the reverse primer TOXO-R (5 μM, 5′-AGCGTTCGTGGTCAACTATCGATTG-3′) (IDT, Coralville, Iowa), and 2.5 μl of TaqMan probe (2 μM, 6FAM-TCTGTGCAACTTTGGTGTATTCGCAG-TAMRA) (Thermo Fischer Scientific, Waltham, MA, United States) in a final volume of 25 μl. The length of expected fragment was 97 bp. The qPCR was run on the GenAmp 7500 Sequence Detection System (Thermo Fischer Scientific, Waltham, MA, United States). After initial activation at 95°C for 10 min, 40 qPCR cycles of 95°C for 15 s and 60°C for 1 min were run. The Ct, indicative of the quantity of target gene at which the fluorescence exceeds a preset threshold, was determined. This threshold was defined as 20 times the standard deviation of the baseline fluorescent signal, i.e., the normalized fluorescent signal of the first few PCR cycles. After reaching the threshold, the sample was considered positive (Lin et al., 2000).

### Semi-Nested PCR for Detection of *Toxoplasma gondii* B1 Gene

DNA detection of *T. gondii* in samples showing a Ct ≥ 35 was confirmed using a semi-nested end-point PCR (Lin et al., 2000) with few modifications. Five microliters of template DNA was added to a final volume of 25 μl of PCR mixture consisting of 12.5 μl of 2x KAPA Hotstart Ready Mix PCR Kit (Kapa Biosystems Inc., Wilmington, Massachusetts), 1.5 μl of 10 μM forward primer (TOXO1; 5′-GGAACTGCATCCGTTCSTGAG-3′) (IDT, Coralville, Iowa), and 1.5 μl of 10 μM reverse primer (TOXO2; 5′-TCTTTAAAGCGTTCGTGGTC-3′) (IDT, Coralville, IA, United States). The mixture was denatured at 95°C for 5 min, followed by 40 cycles of 95°C for 30 s, 60°C for 30 s, 72°C for 30 s, and final elongation at 72°C for 1 min. All the PCR products were analyzed using the Tape Station 2200, an automated platform for electrophoresis (Agilent Technologies, Santa Clara, CA, United States) using the D1000 screen tape system following manufacture’s protocol. When samples showed low electrophoretic band density, 5 μl of the amplicon were re-amplified (semi-nested PCR) under identical conditions in a reaction mixture where the primer TOXO1 was replaced by the primer TOXO4 (5′-TGCATAGGTTGCAGTCACTG-3′) (IDT, Coralville, IA, United States) (Lin et al., 2000).

### Genotype Analysis of *Toxoplasma gondii* Isolates

Genetic characterization of *T. gondii* isolates was performed by sequencing at 5 microsatellite markers (TUB2, W35, TgM-A, B18, and B17) (Thermo Fischer Scientific Waltham Massachusetts) using a multiplex PCR assay (Ajzenberg et al., 2005). Briefly, PCR was carried out in a 25 μl reaction mixture consisting of 12.5 μl of 2X Qiagen Multiplex PCR Master Mix (Qiagen, Hilden, Germany), 5 μl Q solution 1X final concentration and 0.04 μM of each primer. The volumes of DNA template were 5 μl of DNA (1 pg/μl) extracted from positive controls and 5 μl for DNA extracted directly from mussels samples. RH Type I (ATCC: 50174D), PTG strain Type II (ATCC: 50841), CTG Type III (ATCC: 50842), and MAS atypical strain (ATCC: 50870), already genotyped elsewhere (Ajzenberg et al., 2010) were used in this study as positive controls. Amplification was carried out in Mastercycler Nexus X2 (Eppendorf, Hamburg, Germany) and consisted of an initial denaturation at 95°C for 15 min, followed by 45 cycles consisting of 94°C for 30 s, 55°C for 3 min and 72°C for 60 s. The last extension step was made at 60°C for 30 min. One microliter of PCR product was mixed with 0.3 μl of LIZ 500 Size standard and 13.7 μl of HI-Di Formamide. The mixture was denatured for 5 min at 95°C and analyzed by capillary electrophoresis with an ABI PRISM 3500 Genetic Analyzer (Thermo Fischer Scientific, Waltham, MA, United States), equipped with a 50 cm long capillary filled with the separation medium POP-7. Microsatellite fragment analysis was performed by Gene MapperTM v. 3.7. The minimum fluorescence threshold for valid peaks was set at 50 RFU. Low peaks were confirmed by repeating the PCR amplification with a single primer pair. The variability of the Short Tandem Repeats (STRs) of the *Toxoplasma* strains included in this study was evaluated by hierarchical cluster analysis using average method^2^ (Wessa, 2009). Analysis included also genotypes of reference strains from published literature (Ajzenberg et al., 2010).

## Results

*Toxoplasma gondii* DNA was detected in 39 out of 382 (10.2%) Mediterranean mussels from six out of seven sampling sites in the Gulf of Naples, and in four out of 27 individuals from the mollusk depuration plant in Corigliano Calabro. The prevalence of infection ranged from 0 to 21.6% depending on the sampling site (Table 1), with Torre del Greco as the only *T. gondii* negative sampling site. In general, the minimum and the maximum number of *T. gondii* copies/g in the digestive gland were 0.14 from Lucrino and 1.18 from Torre Annunziata, respectively. The mean number of oocysts per gram of the digestive gland ranged from 1.37 to 2.05.

**TABLE 1:** Collection sites and parasite load of *T. gondii* DNA in Mediterranean mussels (*Mytilus galloprovincialis*).

**Collection site**	**Mussels (*N*)**	**Positive (*N*)**	**Prevalence (%)**	**Minimum value (copies/g)**	**Maximum value (copies/g)**	**Mean value (copies/g)**
Mergellina	60	13	21.6	0.72	1.16	0.9
Torre Annunziata	60	9	15	0.53	1.18	0.8
Lucrino	90	11	12.2	0.15	1.14	0.8
Monte di Procida	17	2	11.7	0.42	1.10	0.76
Ercolano	30	2	6.6	0.15	1.10	0.6
Capo Miseno	95	2	2.1	0.49	1.12	0.8
Torre del Greco	30	0	0	0	0	0
Corigliano Calabro (mollusk depuration plant)	27	4	14.8	0.61	1.19	0.9
**Total**	409	43	10.5			

Fragment analysis of STRs at 5 microsatellite loci (TUB2, W35, TgM-A, B18, and B17) (Table 2) was performed from 10 *T. gondii* multiplex PCR positive mussels representative of four out of seven of those sampling sites where *T. gondii* DNA was detected. These included four isolates from Lucrino, three isolates from Corigliano Calabro, two isolates from Monte di Procida and one isolate from Capo Miseno.

**TABLE 2:** Microsatellite markers and PCR primers used as previously described by Ajzenberg et al. (2005).

**Marker**	**Coding function**	**Primers sequences**
TUB2 (1X)	Beta-tubulin gene	(F) 5′ 6FAM-GTCCGGGTGTTCCTACAAA 3′ (R) 5′ TTGGCCAAAGACGAAGTTGT
W 35 (II)	Unknown (EST)	(F) 5′ GGTTCACTGGATCTTCTCCAA 3′ (R) 5′ 6FAM-AATGAACGTCGCTTGTTTCC 3′
TgM-A (X)	Myosin A gene	(F) 5′ GGCGTCGACATGAGTTTCTC 3′ (R) 5′ HEX-TGGGCATGTAAATGTAGAGATG 3′
B 18 (VII)	Unknown (EST)	(F) 6FAM-TGGTCTTCACCCTTTCATCC 3′ (R) 5′ AGGGATAAGTTTCTTCACAACGA 3′
B 17 (XII)	Unknown (EST)	(F) 5′ AACAGACACCCGATGCCTAC 3′ (R) 5′ HEX-GGCAACAGGAGGTAGAGGAG 3′

*EST, expressed sequence tag; F, forward primer; R, reverse primer.*

Microsatellite analysis revealed the presence of 5 distinct genotypes (Table 3). Six samples displayed the same genotype corresponding to type I (27379_A, 27379_B, 66471_7, 70259_1, 41680_B, and 86033_9) including two isolates each from Monte di Procida and Lucrino, and one isolate each from Capo Miseno and Corigliano Calabro. Four samples (two from Corigliano Calabro and two from Lucrino) displayed atypical genotypes, one of which (sample 44825_C) corresponding to the previously characterized Atypical – CAST genotype (Ajzenberg et al., 2010).

**TABLE 3:** Genotyping results of 10 field strains and 26 reference strains with 5 STRs markers.

**Sample Name**	**TUB2**	**W35**	**B18**	**B17**	**TgM-A**	**Sampling site**	**Genotype**	**References**
27379_A	291	248	160	342	209	Monte di Procida	GI	This study
27379_B	291	248	160	342	209	Monte di Procida	GI	This study
66471_2	289	246	158	342	207	Lucrino	Atypical	This study
66471_7	291	248	160	342	209	Lucrino	GI	This study
70259_1	291	248	160	342	209	Capo Miseno	GI	This study
G I – RH	291	248	160	342	209	ATCC	–	This study
G II – PTG	289	242	158	336	207	ATCC	–	This study; Ajzenberg et al., 2010
G III – CTG	289	242	160	336	205	ATCC	–	This study; Ajzenberg et al., 2010
Atypical – MAS	291	242	162	362	205	ATCC	–	This study; Ajzenberg et al., 2010
G II – NTE	289	242	158	336	207	–	–	Ajzenberg et al., 2010
G III – NED	289	242	160	336	205	–	–	Ajzenberg et al., 2010
Atypical – CASTELLS	287	242	158	358	207	–	–	Ajzenberg et al., 2010
G I – CT1	291	248	160	342	209	–	–	Ajzenberg et al., 2010
G I – GIL	291	248	160	342	209	–	–	Ajzenberg et al., 2010
G III – M7741	289	242	160	336	205	–	–	Ajzenberg et al., 2010
Atypical – TgCatBr1	289	242	160	342	205	–	–	Ajzenberg et al., 2010
G II – BOU	289	242	158	336	207	–	–	Ajzenberg et al., 2010
G I – BK	291	248	160	342	209	–	–	Ajzenberg et al., 2010
41680_B	291	248	160	342	209	Corigliano Calabro	GI	this study
44035_C	289	242	160	336	203	Corigliano Calabro	Atypical	this study
44825_C	291	242	158	342	205	Corigliano Calabro	Atypical - CAST	this study
86033_29	291	248	160	342	209	Lucrino	GI	this study
86033_15	291	242	162	336	205	Lucrino	Atypical	this study
G III – VEG	289	242	160	336	205	–	–	Ajzenberg et al., 2010
Atypical – GUY-COE	289	246	160	337	203	–	–	Ajzenberg et al., 2010
Atypical – GUY-MAT	291	242	160	339	203	–	–	Ajzenberg et al., 2010
Atypical – RUB	289	242	16	360	205	–	–	Ajzenberg et al., 2010
Atypical – GPHT	291	248	160	342	205	–	–	Ajzenberg et al., 2010
Atypical – BOF	291	248	160	342	205	–	–	Ajzenberg et al., 2010
Atypical – CAST	291	242	158	342	205	–	–	Ajzenberg et al., 2010
Atypical – TgCaBr5	291	242	160	362	205	–	–	Ajzenberg et al., 2010
Atypical – P89	291	242	160	348	205	–	–	Ajzenberg et al., 2010
Atypical – TGCaBr3	289	242	160	348	205	–	–	Ajzenberg et al., 2010
Atypical – VAND	291	242	162	344	203	–	–	Ajzenberg et al., 2010
Atypical – GUY-DOS	289	246	160	344	203	–	–	Ajzenberg et al., 2010
Atypical – COUGAR	289	242	158	336	205	–	–	Ajzenberg et al., 2010

**FIGURE 2:**
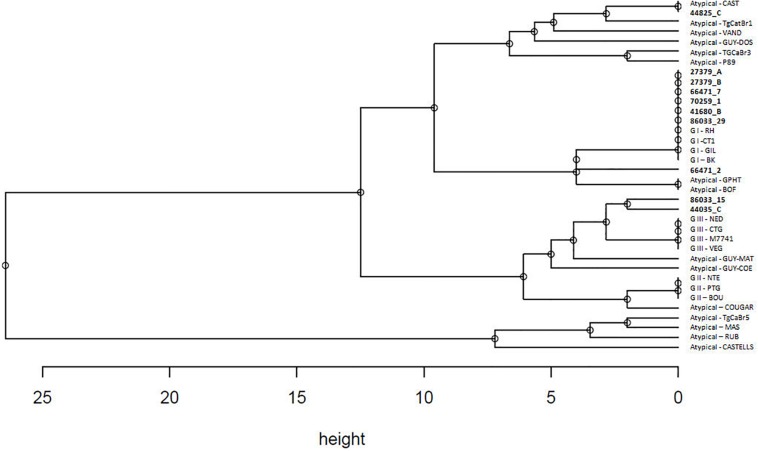
Hierarchical clustering analysis performed with average linkage method based on STRs genotypes of 10 field strains and 26 reference strains as reported in Table 3. Strains detected in the present study are marked in bold.

Hierarchical clusters analysis showed three well distinct clusters (Figure 2), one including GI type, one including GII and GIII types, and one including only atypical isolates. Atypical genotypes appeared variously distributed among clusters. Among the atypical genotypes identified in the present study, the samples 44825_C and 66471_2 (from Corigliano Calabro and Lucrino, respectively) clusterized with the group including GI strains, while the samples 86033_15 and 44035_C (from Lucrino and Corigliano Calabro, respectively) clusterized with the group including GIII strains (Figure 2).

## Discussion

This study reports on the quantitative molecular data and genotyping of *T. gondii* in the Mediterranean mussels from the Mediterranean Sea, and provides the first evidence of new atypical genotypes within the marine environment and marine food chain. This represents the most extensive molecular and genotyping study on *T. gondii* in individual Mediterranean mussels. We used the STRs genotyping because this technique showed a higher sensitivity and power of resolution than PCR-RFLP, and was able to identify higher genetic diversity within *T. gondii* isolates (Ajzenberg et al., 2010; Su et al., 2010; Feitosa et al., 2017). The STRs analysis was here useful to distinguish genetically closely related *T. gondii* strains, revealing the occurrence of 5 distinct genotypes.

Parasite load and genotyping may be a useful way to assess the infection risk for the consumer since *T. gondii* occurrence and severity of infection could depend mainly by parasite forms, doses, genotypes, and immune status of the host (Mead et al., 1999; Pereira et al., 2010; Robert-Gangneux and Dardè, 2012). In the present study, the mean number of oocysts per gram of digestive gland ranged from 1.37 to 2.05. The parasite load detected in the present study is of great health public significance, however, the positivity to the *T. gondii* DNA does not necessarily mean the presence of its viable oocysts in Mediterranean mussels. According to Dubey (1996), a single live oocyst is orally infective to a pig. In southern Italy, the Mediterranean mussels may be consumed uncooked or lightly cooked, and it may strongly increase the risk of *T. gondii* infection for humans.

The overall prevalence (10.2%) here found studying individually 382 Mediterranean mussels from seven sampling sites of the Gulf of Naples appears high, but because of different methods of examination (individual versus pooled mussel examination), it is very hard to compare results here obtained with prevalence of similar studies (Putignani et al., 2011; Aksoy et al., 2014; Tedde et al., 2019). Differences found among sampling sites with prevalence rates ranging from 0 to 21.6% may suggest different levels of seawater contamination by *T. gondii* oocysts depending on few abiotic factors including mainly marine currents and proximity to the freshwater outflow. It has been reported that *T. gondii* detection was significantly higher in California mussels (*Mytilus californianus*) sampled in the wet season and in sampling sites close to the freshwater runoff in California. This finding strongly suggests that *T. gondii* enters the nearshore environment through rain driven contaminated overland runoff (Shapiro et al., 2015).

Data from genotyping assay here obtained reveals the simultaneous presence of multiple *T. gondii* strains at a single sampling site showing, in general, a rich diversity with five different genotypes, 4 (80%) of which are atypical. The richness in strain types here found suggests several potential sources of infection for Mediterranean mussels by felid fecal material into the coast line environment of Gulf of Naples and as well as in the Mediterranean mussels collected from the mollusk depuration plant in Corigliano Calabro. Regarding the isolates from Corigliano Calabro, whether Mediterranean mussels were contaminated with *T. gondii* in the depuration plant or in the northern Italy farm from which mollusks came is unknown.

Most genotypes here found can be related to type I, which is one of the three known main lineages. In Europe the population structure of *T. gondii* is markedly clonal with a predominance of strains belonging to the type II; the type III is occasional, the type I is exceptional, and atypical strains are rare (Robert-Gangneux and Dardè, 2012). Concerning genotyping, the type I has been detected in 5 out of 21 (23.8%) pooled samples of Mediterranean mussels from the Antolian Peninsula (Aksoy et al., 2014). The prevalence of *T. gondii* in California mussels in California was 1.4% (Shapiro et al., 2015). Based on distinct RFLP banding patterns and sequence analysis at the B1 locus, it has been detected *T. gondii* type I in two mussels, types II/III in one mussel, and type X (atypical) in three mussels, with an unique RFLP cleaving pattern detected in two mussels (Shapiro et al., 2015). Atypical genotypes found in California mussels were identical to *T. gondii* strains found in carnivore species (domestic cats, foxes, and mountain lion) from central coastal California (VanWormer et al., 2014; Shapiro et al., 2015), strongly supporting the hypothesis of mussel contamination via freshwater runoff.

Results here obtained, suggest potential implications of epidemiological importance for human health because both type I and atypical genotypes are considered as highly pathogenic, being associated with acquired ocular diseases in patients with disseminated congenital toxoplasmosis (Dardè et al., 2007), and severe acute toxoplasmosis outbreaks (Carme et al., 2009; Vaudaux et al., 2010; Demar et al., 2012), respectively.

The occurrence of *T. gondii* oocysts belonging to potential pathogenic genotypes in the Mediterranean mussels from almost all sampled localities suggests the ubiquity of the parasite in the Gulf of Naples, and it increases the likelihood of infection for all at-risk species in the marine ecosystem, including humans that use marine resources (Miller et al., 2004; Mazzillo et al., 2013; VanWormer et al., 2014; Aguirre et al., 2019). The present data indicate that the presence of these pathogens in southern Italy should be a health concern to people in this region, and an effective ecosystem management approach should include the control of contaminated runoff to mitigate the health impacts of coastal habitat pathogen pollution (Aguirre et al., 2019). Molecular surveillance of shellfish for *T. gondii* may be important for monitoring the marine environmental contamination by oocysts, and predicting and controlling the spread of infection for humans and wildlife (Miller et al., 2004; Mazzillo et al., 2013; VanWormer et al., 2014; Aguirre et al., 2019).

## Data Availability Statement

The raw data supporting the conclusions of this article will be made available by the authors, without undue reservation, to any qualified researcher.

## Ethics Statement

The Istituto Zooprofilattico Sperimentale del Mezzogiorno is accredited by the Italian Ministry of Health to perform systematic surveys on infectious diseases of veterinary importance. Procedures for this study were performed in accordance with the guide for the care and use of aquatic organisms by the Italian Ministry of Health.

## Author Contributions

MS, AA, VV, GG, LB, and GF conceived the study. MV, FB, GB, ML, and CA performed the molecular and genotyping analysis. MS wrote the manuscript. All authors reviewed and approved the final manuscript.

## Conflict of Interest

The authors declare that the research was conducted in the absence of any commercial or financial relationships that could be construed as a potential conflict of interest.

## Footnotes

1 http://www.endmemo.com/bio/dnacopynum.php

2 https://www.wessa.net/rwasp_hierarchicalclustering.wasp/
